# Proton pump inhibitors and dementia: A nationwide population‐based study

**DOI:** 10.1002/alz.13657

**Published:** 2024-01-06

**Authors:** Jinmiao Lu, Yi Wang, Zhiping Li

**Affiliations:** ^1^ Department of Pharmacy Children's Hospital of Fudan University National Children's Medical Center Minhang Shanghai China; ^2^ Department of Neurology Children's Hospital of Fudan University National Children's Medical Center Minhang Shanghai China

Dear editor,

We have read the manuscript “Proton pump inhibitors and dementia: A nationwide population‐based study” by Pourhadi N et al.^1^ The study found that proton‐pump inhibitor (PPI) use was associated with an increased risk of Alzheimer's disease (AD), although the cause‐and‐effect relationship remains unclear. PPIs are commonly used in adults to treat acid‐related disorders such as peptic ulcer and acid reflux. However, over 40% of patients treated with PPIs fail to control their symptoms effectively. In addition to PPIs, treatments for acid‐related disorders often include drugs containing aluminum (Al). Al‐containing preparations are widely used in the adjuvant treatment of gastrointestinal diseases, with antacid and protective gastric mucosa of special efficacy, rarely discontinuation.^2^ Humans' daily Al intake can be as high as 5000 mg if sucralfate (a preparation containing Al) is used at the recommended dose.^3^ Therefore, we speculate that this study may contain an undiscovered association between Al preparations and AD.

The relationship between Al and AD remains an ongoing century‐long debate.^4^ In vivo, Al is an experimental neurotoxin. Many classic animal models of AD can be established by injection or oral Al compounds.^5^ There also exists an association between Al and AD and long‐term exposure to high concentrations, with older animals being more sensitive to Al neurotoxicity than younger animals.^6^ Among humans, Al exposure has been associated with various neurodegenerative conditions, including amyotrophic lateral sclerosis, Parkinson's disease dementia, and AD.^7^ Some investigations have suggested a potential link between prolonged Al exposure and AD, while others have failed to corroborate this connection, yielding inconclusive findings. Until now, definitive evidence establishing a direct association between Al and AD has not materialized. Historically, in pediatric patients with kidney disease, prolonged use of Al‐containing dialysis solutions has led to the accumulation of Al and dementia, referred to as dialysis encephalopathy.^6^ The shared clinical features between dialysis encephalopathy and AD have prompted the hypothesis that the mechanism through which Al triggers dementia may be interrelated with the renal system's inability to metabolize Al. Clinical trials have shown that the average plasma Al concentration of healthy volunteers over 65 increases by two to three times after receiving Al‐containing preparations.^8^ Thus, we hypothesize that reduced renal excretion of Al in older adults and continued consumption of Al‐containing products may ultimately contribute to AD development. An association between Al and AD has recently been revisited.^9^

A new epidemiological study showed that esophageal reflux was significantly associated with the risk of AD.^10^ The curious result is that PPI (esomeprazole/pantoprazole) is associated with a significantly lower incidence of AD. This result contradicts the PPI‐AD‐related conclusion. Therefore, we consider that exposure to Al‐containing antacids in PPI consumers may affect the data provided. We suggest that the author consider further investigating the correlation between the use of Al antacids and AD in future papers.

In summary, Pourhadi N et al. did not report whether individuals using PPIs also concurrently used Al‐containing preparations. The lack of these data may introduce a potential study bias. However, this study is the most extensive follow‐up investigation, spanning a median follow‐up duration of 10 years. Based on these findings, we can establish a link between gastrointestinal conditions and AD, with a more pronounced correlation observed when the diagnosis occurs at a younger age.

## CONFLICT OF INTEREST STATEMENT

The authors declare no conflicts of interest. Author disclosures are available in the supporting information.

## Supporting information

Supporting Information

## References

[1] Pourhadi N , Janbek J , Jensen‐Dahm C , Gasse C , Laursen TM , Waldemar G . Proton pump inhibitors and dementia: a nationwide population‐based study. Alzheimers Dement. 2023. Online ahead of print. 10.1002/alz.13477 PMC10917029 37795826

[2] McCarthy DM . Sucralfate. N Engl J Med. 1991;325:1017‐1025. 1886624 10.1056/NEJM199110033251407

[3] Lione A . Aluminum intake from non‐prescription drugs and sucralfate. Gen Pharmacol. 1985;16:223‐228. 3839482 10.1016/0306-3623(85)90073-4

[4] Tomljenovic L . Aluminum and Alzheimer's disease: after a century of controversy, is there a plausible link? J Alzheimer's Dis. 2011;23:567‐598. 21157018 10.3233/JAD-2010-101494

[5] Song J . Animal model of aluminum‐induced Alzheimer's disease. Neurotoxicity of Aluminum: Springer; 2023:123‐138.

[6] Corkins MR , Abrams SA , Fuchs GJ , et al. Aluminum effects in infants and children. Pediatrics. 2019;144:e20193148. 31767714 10.1542/peds.2019-3148

[7] Walton J . Chronic aluminum intake causes Alzheimer's disease: applying Sir Austin Bradford Hill's causality criteria. J Alzheimer's Dis. 2014;40:765‐838. 24577474 10.3233/JAD-132204

[8] Moore JG , Coburn JW , Sanders MC , McSorley DJ , Sirgo MA . Effects of sucralfate and ranitidine on aluminum concentrations in elderly volunteers. Pharmacotherapy. 1995;15:742‐746. 8602382

[9] Niu Q . Overview of the relationship between aluminum exposure and human health. Neurotoxicity of Aluminum: Springer; 2023:1‐32. 10.1007/978-981-13-1370-7_1 30315446

[10] Fan P , Miranda O , Qi X , Kofler J , Sweet RA , Wang L . Unveiling the enigma: exploring risk factors and mechanisms for psychotic symptoms in Alzheimer's disease through electronic medical records with deep learning models. Pharmaceuticals. 2023;16:911. 37513822 10.3390/ph16070911 PMC10385983

